# Broad-scale morpho-functional traits of the mandible suggest no hard food adaptation in the hominin lineage

**DOI:** 10.1038/s41598-020-63739-5

**Published:** 2020-04-22

**Authors:** Jordi Marcé-Nogué, Thomas A. Püschel, Alexander Daasch, Thomas M. Kaiser

**Affiliations:** 1 Centrum für Naturkunde, University of Hamburg, Martin-Luter-King-Platz 3, 20146 Hamburg, Germany; 2 Institut Català de Paleontologia M. Crusafont, Universitat Autònoma de Barcelona, Cerdanyola del Vallès, Barcelona, 08193 Spain; 3 Primate Models for Behavioural Evolution, Institute of Cognitive and Evolutionary Anthropology, University of Oxford, 64 Banbury Road, Oxford, OX2 6PN United Kingdom

**Keywords:** Biological anthropology, Biomechanics

## Abstract

An on-going debate concerning the dietary adaptations of archaic hominins and early *Homo* has been fuelled by contradictory inferences obtained using different methodologies. This work presents an extensive comparative sample of 30 extant primate species that was assembled to perform a morpho-functional comparison of these taxa with 12 models corresponding to eight fossil hominin species. Finite Element Analysis and Geometric Morphometrics were employed to analyse chewing biomechanics and mandible morphology to, firstly, establish the variation of this clade, secondly, relate stress and shape variables, and finally, to classify fossil individuals into broad ingesta related hardness categories using a support vector machine algorithm. Our results suggest that some hominins previously assigned as hard food consumers (e.g. the members of the *Paranthropus* clade) in fact seem to rely more strongly on soft foods, which is consistent with most recent studies using either microwear or stable isotope analyses. By analysing morphometric and stress results in the context of the comparative framework, we conclude that in the hominin clade there were probably no hard-food specialists. Nonetheless, the biomechanical ability to comminute harder items, if required as fallback option, adds to their strategy of increased flexibility.

## Introduction

Diet is one of the principal factors underlying the behavioural and ecological differences among living primates. Consequently, primate diets have been more carefully documented than many other aspects of their behaviour^1^. The relationship between ingesta and morphology has been investigated in extant and fossil primates over the last years by applying an array of different techniques. These include biomechanics and comparative morphology^2^, dental wear texture^3^, dental morphology and allometry^4^, as well as stable isotope analysis^5^. Investigating functional morphology questions using Finite Element Analysis (FEA) or Geometric Morphometrics (GMM) is now a standard procedure that has been applied to a variety of vertebrates, thus shedding light on relationship between form and function (see Polly *et al*.^6^ for a review). These techniques have also been applied to study mastication in extant hominins and primates^7^ by analysing both their upper and lower jaws^8^.

Archaic hominins have been shown to display an impressive suite of derived craniodental traits that are widely considered adaptive for feeding^9^. It has been proposed that their rapidly flat worn molars were adapted for breaking down brittle and hard foods, including nuts and some fruits but were not particularly useful for breaking down tough pliant foods such as for example meat, soft seed pods or stems^9^. Nevertheless, contradictory results have emerged, for example, an analysis of *A. afarensis* showed that apparently there is no evidence that hard objects were habitually processed with the premolars or consumed as part of their regular diet^10^. The facial skeleton of *A. africanus* seems well adapted to withstand loads imposed by the ingestion of large sized, mechanically protected objects like large nuts and seeds, which had to be cracked open in the first place using the premolars^11^. However, microwear studies have not confirmed this interpretation, but rather indicated a softer diet^10,12^. The cranium of *A. sediba* was not mechanically optimized to produce high molar bite force and the taxon appears to have been constrained in its capacity to consume hard foods^13^, and also *A. sediba*’s dental microwear analyses have provided contradictory evidence^14^.

It has been proposed that after on-going environmental changes, the broader dietary niche of *Australopithecus* apparently split around 4 Ma and subsequently was occupied by the two more specialized genera *Paranthropus* and *Homo*, which were possibly processing food more efficiently in each one of their respective dietary niches^15–17^. The masticatory apparatus of *Paranthropus* is characterized by its robusticity, enabling exceptional bite force^2^ and traditionally has been considered adapted for harder and more resilient components^18^. However, more recently,  dental microwear and isotopic analyses contradict this notion^3,19^ and seasonal availability and fall back resources may explain some of these discrepancies^3,20^. Nonetheless, contradictory evidence also arose when microwear comparisons showed that the toughness of the ingesta of *P. boisei* ranged within the values obtained for *A. africanus*, even though they did not consume harder and more brittle foods than *P. robustus*^21^.

In *Homo*, there is more consensus among different methodologies, which suggests that the early member of this genus have evolved into a more generalist niche and complement their diet by novel and abundant savannah resources, like ungulate meat and tubers^22^. Earliest African *Homo* individuals assigned to *H. habilis, H. rudolfensis* and *H. ergaster* show derived characters such as a reduction in molar size and enamel thickness, increase in dental topography, steepness of cusp slopes and increase of occlusal relief. These characters are consistent with an increase of sheer-cutting capabilities over crushing action and suggest adaptive traits towards the consumption of animal soft tissue^4^. The Dmanisi hominins^23^, which are roughly contemporaneous with early African *Homo* show microwear patterns suggesting no consumption of foods which were particularly tough or hard^24,25^.

Despite their relevance, to date, cranial biomechanics using FEA have only been studied in *P. boisei*^8,16,26^, *A. africanus*^8,27,28^ and *A. sediba*^13^. Moreover, FEA research on extant primates has mostly focused on the biomechanics of the cranium^29^ rather than the mandible. It has been noted that cranial shape reflects a compromise between different functional demands (e.g., feeding, respiration, phonation, cognition, among others), which could conceal a strong dietary signal^30^, whilst the mandible is predominantly involved in mastication and food acquisition. Accordingly, its morphology would be expected to better reflect ingesta-related activities as compared to the cranium^31^.

Previous works in fossil hominins using both classical^2^ and computational biomechanical approaches^11^ have suggested that hard food consumption caused adaptive responses. However, these studies were based on a small comparative framework. Given the evidence stated above there is an on-going debate concerning regular diets, fallback foods and physical adaptations and transformations related to these traits in fossil hominins. The debate has been partially fuelled by the contradictory results obtained using different methodologies such as microwear/microtexture, stable isotopes or functional morphology that provide fundamentally different and partly non-independent levels and scales of diet related sources of evidence. A comparative assessment of the biomechanics of the craniomandibular apparatus in extant primates would therefore fill a gap in our data, by adding the functional aspect of structure that can be accessed via morphology. The resulting functional framework will allow us testing of ingesta related hypothesis also in the fossil record. Since the mandible is both, crucial in any understanding of ingesta related adaptive trait in primates^8^, as well as most frequently preserved in the fossil record, it is reasonable to base this work on mandibular FEA models. A novelty of our study is the large comparative FEA framework of 30 extant primate species. Along with this, we compare the biomechanical traits of 12 FEA models representing fossil hominin individuals, including eight species of archaic hominins and *Homo*. In addition, geometric morphometrics (GM) were applied to establish morphological variation and to relate stress and shape variables. We thus propose to contextualize the debate on hard vs. soft diets employing extant primate mandibular biomechanics^32^ to classify fossil hominin specimens within biomechanically defined dietary trait patterns.

## Material and Methods

### Sample

A planar stress analysis was performed here^33^. This means that the analysed structural elements have two dimensions larger than another one (i.e. thickness), thus the stresses are negligible with respect to the smaller dimension. The thicknesses of each one of the mandibles under analysis were modelled as constant and were computed as the mean value of three measurements obtained from different mandibular areas. This approach has been successfully applied in previous works analysing mandibles^32,34^ and represents a good alternative when 3D models are not easily available. Thirty extant primate species were previously analysed in Marcé-Nogué *et al*.^32^ and were included in the present study. Primate species were classified according to the relative toughness of their typical food into two categories: soft-food and hard-food eaters as described in Marcé-Nogué *et al*.^32^ (Table 1). We are aware that any classification scheme corresponds to a simplification of the feeding behaviour of the animals under study, because any discretization always implies some error as variability is reduced into a limited number of categories. Nevertheless, our binary classification scheme is based on previous publications that classified feeding behaviour based on the material properties of the main ingesta of the analysed species. Hence it should be a good representation of a general -but important- dietary characteristic. Additionally, 12 models representing eight fossil hominin species were also analysed (Table 2).

**Table 1:** List of primate species used in the present study.

SPECIE	Accession number	FAMILY	TRAIT
*Alouatta seniculus*	ZMH-S 3495	Atelidae	S
*Aotus trivirgatus*	ZMH-S 5276	Cebidae	H
*Ateles geoffroyi*	ZMH-S 2994	Atelidae	H
*Brachyteles arachnoides*	ZMB-Mam- 36455	Atelidae	H
*Callithrix jacchus*	ZMH-S 3299	Cebidae	H
*Cebus apella*	ZMH-S 3567	Cebidae	H
*Cebus capucinus*	ZMH-S 3950	Cebidae	H
*Cercocebus torquatus*	ZMH-S 6381	Cercopithecoidea	H
*Chlorocebus aethiops*	ZMH-S 4555	Cercopithecoidea	S
*Eulemur fulvus*	ZMB-Mam- 7768	Lemuridae	S
*Gorilla gorilla*	ZMH-S 6992	Hominoidae	S
*Hapalemur griseus*	ZMB 35263	Lemuridae	S
*Homo sapiens*	ZMH-S 9537	Hominoidae	S
*Hylobates lar*	ZMH-S 7013	Hylobatidae	S
*Hylobates moloch*	ZMH-S 8369	Hylobatidae	S
*Hylobates muelleri*	ZMB-Mam- 7863	Hylobatidae	S
*Lemur catta*	ZMH-S 3259	Lemuridae	S
*Macaca fascicularis*	ZMH-S 10191	Cercopithecoidea	S
*Macaca fuscata*	ZMH-S 9495	Cercopithecoidea	H
*Macaca mulatta*	ZMH-S 4755	Cercopithecoidea	H
*Macaca nemestrina*	ZMH-S 3274	Cercopithecoidea	H
*Nycticebus coucang*	ZMH-S 4807	Lorisidae	H
*Pan troglodytes*	ZMH-S 2756	Hominoidae	S
*Papio cynocephalus*	ZMH-S 6802	Cercopithecoidea	H
*Papio ursinus*	ZMB-Mam- 18047	Cercopithecoidea	H
*Pithecia pithecia*	ZMH-S 7625	Pitheciidae	H
*Pongo pygmaeus*	ZMH-S 9395	Hominoidae	H
*Saimiri sciureus*	ZMH-S 7633	Cebidae	H
*Therophitecus gelada*	ZMH-S 3273	Cercopithecoidea	S
*Trachypithecus cristatus*	ZMH-S 1818	Cercopithecoidea	S

Museum acronyms: ZMH = Centrum für Naturkunde, Hamburg, Germany; ZMB = Museum für Naturkunde Berlin, Germany. Families are according the classification of Arnold *et al*. 2010 and Wilson and Reeder, 2005^35,36^. H: Hard-foot eaters and S: Soft-food eaters.

**Table 2:** List of fossil hominins used in the present study.

ID	Accession number	GENUS
*Au. afarensis 1*	reconstruction^a^	*Australopithecus*
*Au. afarensis 2*	AL 444-2	*Australopithecus*
*Au. afarensis 3*	AL 822-1	*Australopithecus*
*Au. africanus 1*	Sts 36	*Australopithecus*
*Au. africanus 2*	Sts 52^b^	*Australopithecus*
*Au. sediba*	UW 88-8	*Australopithecus*
*P. robustus*	DNH7	*Paranthropus*
*P. boisei*	Peninj1	*Paranthropus*
*H. rudolfensis*	KNM-ER 60000	*Homo*
Georgian *H. erectus 2*	D2735	*Homo*
Georgian *H. erectus 5*	D2600	*Homo*
Asian *H. erectus*	reconstruction^c^	*Homo*

^a^The reconstruction of Au. afarensis 1 is based on Kimbel *et al*.^37^. ^b^The reconstruction of Au. Africanus 2 in Benazzi *et al*.^38^. ^c^The reconstruction of Zhoukoudian, Loc 1 male H. erectus is based on Tattersall and Sawyer, 1996^39^.

### Reconstruction of the models

The mandibles were analysed as planar 2D models using the FEA software ANSYS® (Ansys Inc., v.17.1, Canonsburg, PA, USA; http://www.ansys.com/). The different steps described in Marcé-Nogué *et al*.^32^ were followed in order to generate digitals models from pictures. This approach generates the models from lateral pictures of the different taxa. The photos of the extant species were taken from the original material. In the case of the fossil taxa, the same procedure was followed with a previous reconstruction of the models. Fossil taxa reconstructions are described in the Supplementary Information.

The elements used to mesh the FEA models of the different mandibles corresponds to 8-node quadrilateral plane elements (QUAD8). This allowed us creating the quasi-ideal mesh (QIM) proposed by Marcé-Nogué *et al*.^40^, which corresponds to a mesh with enough density to properly represent variation in stress patterns, hence ensuring stable numerical results when considering that a high-quality mesh should have a high level of homogeneity in the size of its elements. This procedure was carried out to guarantee that the subsequent statistical analyses were not affected by the size differences of the element in the mesh. The number of elements of each model can be found in Table S1 in SI.

The thickness of the model was modelled as constant throughout the mandible. This value was computed from the individual average of three measurements in the extant primates. THK1: mandibular breadth at the first premolar, THK2: mandibular breadth at the mid-point of the mesio-distal length of the molar and premolar series and THK3: mandibular breadth at the posterior end of the molar series. Thickness values were measured directly by JMN on the extant specimens and on the casts of the fossil taxa. Missing values for the remaining specimens were collected from diverse publications, measured at similar positions (Table S1 in SI shows the thickness used in each model and the source). Isotropic, homogeneous and linear elastic properties were used based on data from a *Macaca* mandible: E [Young’s modulus] p: =21 GPa and v [Poisson ratio] = 0.45^41^, even if it has been shown that this value is not crucial in a comparative analysis such as the one carried out here^42^.

### Bite conditions and muscle forces

**Figure 1:**
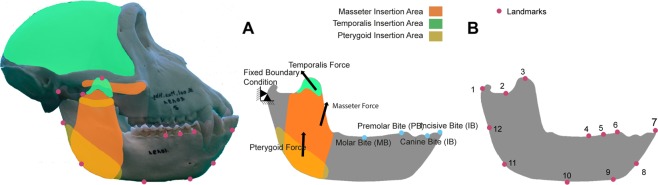
(**A**) Free-Body Diagram of *M. fascicularis* showing the biomechanical scenario, the boundary conditions, the muscular forces, the area of insertion and the Bite position during Incisive Bite (IB), canine Bite (CB), Premolar Bite (PB) and Molar Bite (MB). (**B**) Landmark configuration for the GMM Analysis.

Boundary conditions were modelled to characterize the fixed displacements and loads that the mandibles experiences during feeding. The most posterior point of the condyle at the level of the contact points with the mandibular fossa of the cranium was used as the first boundary condition (Fig. 1). The second boundary condition was applied to simulate biting being placed at four different tooth positions, which correspond to four different scenarios (1) IB (incisive bite): At the buccal alveolar margin of the incisive. (2) CB (canine-bite): At the centre of the canine at the level of the alveolar margin (3) PB (pre-molar bite): Between the most distal Premolar and the first Molar at the level of the alveolar margin. (4) MB (molar bite): At the centre of M1.

The forces applied in the FEA models were scaled using the quasi-homothetic transformation proposed by Marcé-Nogué *et al*.^43^ to enable reasonable comparisons between models due to their size differences. We were not interested in the *in vivo* force values or to validate our results against experimental data, but we rather carried out a comparative analysis^34^. Hence, scaling the forces enables a meaningful comparison between stress results. *H. sapiens* was used as a reference model with a value of F = 1 N. The value of the total force was distributed between the masseter, the temporalis and the pterygoid based on the insertion area of these muscles on the cranium and mandible. For the rest of the models under study, a proportional force based on their size differences was calculated, which assumed that the muscle attachment is proportional to the muscle force (Tables S2 and S3 in SI). The vector directions of each muscle were estimated using the area centroid of the muscle attachment areas.

### Analysis of von mises stress

Von Mises stress distribution is the most adequate standard for predicting the yield of a ductile materials when isotropic material properties are assumed in cortical bone^44^. Quantitative measurements of the relative strength of the structure under study were preferred to summarize the strength of the whole model. Here we used quasi-ideal meshes (QIM) and their percentile values (M25, M50, M75 and M95)^40^. The use of a QIM mesh contributed to the comparison between models and it included the corrections to account for the non-uniformity of the mesh.

All statistical analyses were performed in R v. 3.4.4 (https://www.R-project.org/). Pairwise PERMANOVA tests with a Holm correction for multiple comparisons were performed to assess if there were differences in stress values between the different categories. Euclidean distances were used as similarity index in all tests.

### Geometric morphometrics

Landmark collection was carried out by one of us (JMN) using the R package “geomorph”^45^. Twelve two-dimensional points were collected along the mandibles outlines to characterize mandibular variation (Fig. 1). GM and statistical analysis were performed using the same package. A generalized Procrustes analysis was carried out to calculate shape residuals, by removing all the differences due to scale, translation and rotation. A principal component analysis (PCA) of the shape variables was carried out to display the main axes of variation. The phylogenetic signal of the shape data was computed using a version of the K-statistic appropriate for multivariate data (i.e., Kmult^46^). The associations between mandibular shape (i.e., Procrustes coordinates), and mandibular strength (i.e. stress percentile values: M25, M50, M75, M95 for the four biting scenarios) was analysed using a phylogenetic partial least squares (PLS) analysis^47^. The Phylogenetic PLS computes the level of covariation between the two blocks of data while also taking into account the phylogenetic structuration of the data by assuming a Brownian motion evolutionary model^48^.

### Food hardness classification

We wanted to test whether it was possible to distinguish between food hardness categories using biomechanical stress data obtained from the FEA scenarios and the shape variables derived from GM, as well as to test if it is possible to confidently classify the fossil sample into one of these categories to reconstruct some of their dietary aspects. Therefore, the dataset was analysed and used to classify the fossil taxa based on the categories provided in Table 1. The biomechanical data comprised all the percentiles representing the four tested biomechanical scenarios. Prior to the analysis, a Box-Cox transformation was performed to normalize the percentile data. In addition, these percentiles were centred and scaled to improve the numerical stability and to standardize their scale. This resulted in variables with a zero mean and a common standard deviation of one. These transformed percentiles were then used in the classification analyses.

The morphometric data comprised the number of PCs that accounted for ca. 95% of the sample variance. This provided ten PCs that accounted for 95.83% of the total variance of the sample. Since the original raw coordinates were subjected to a Procrustes superimposition, there was no need to perform any pre-processing procedure prior to the application of the ML classification methods. Additionally, because a PCA was carried out using these shape variables, we avoided any collinearity.

Two support vector machine (SVM) models were trained using the extant data to then be used to classify the fossil sample. One model was trained using the biomechanical data, whilst the other one used the morphometric information. SVMs correspond to a group of related learning methods for classification and regression, which are considered to be among the most powerful and flexible modelling techniques. We preferred to use SVMs instead of more traditional approaches such as linear discriminant analysis or its extension canonical variate analysis because these latter approaches assume multivariate normality, require more specimens than variables (i.e., the pooled within-group variance-covariance matrix needs to be fully ranked to allow matrix inversion), and also because more sophisticated classification techniques might exhibit better discriminating performances^49^.

The models were generated using the ‘caret’ package for R^50^. We decided to train SVM models using a linear kernel for simplicity. The ‘caret’ package also provides a grid search where it is possible to specify tuning parameters for the models. We first started with an automatic grid search. Then the most accurate model was further tuned by setting a manual grid search. In the grid, each algorithm parameter was specified as a vector of possible values, which in this case consisted exclusively of ‘cost’ values, because we were applying a linear kernel. Then using the best final model, the fossil sample was classified into the different food hardness categories by computing the class probabilities of belonging to each one the categories.

The performance of the classification models was quantified using the confusion matrix from which the overall classification accuracy was calculated. In addition, Cohen’s Kappa was also calculated as a performance measurement^51^. To assess the performance of the models, a leave-one-subject-out cross validation was used. Further details about the applied SVM can be found in the SI.

## Results

### Von mises stress distribution

**Figure 2:**
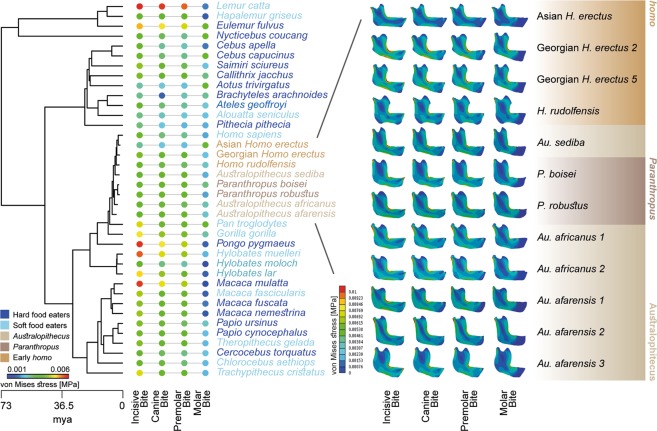
Values of the percentile M50 of the von Mises stress for both extinct and extant species. A composite phylogeny based on the dated consensus tree from version 3 of 10Ktrees^36^ and the latest phylogeny for fossil hominins from^52^ was generated. This phylogeny was slightly modified to remove species for which there were no biomechanical data. Von Mises Stress distribution for each fossil specimen under the four different bite cases are also included: IB: incisive bite; CB: canine bite; PB: premolar bite and MB: molar bite.

All fossil mandibles displayed high levels of stress at the mandibular notch, and from the condyle through the ramus in a descending direction (Fig. 2). This behaviour is quite similar to what is observed in the extant primates^32^ (Fig. S1 in S1). Figure 2 shows the intensity of the value of the percentile M50 in all the models (both actual and fossil) and Figure S2 in SI shows the stress distribution of the QIM in boxplots. Values of percentile stresses can be found in Tables S4–S7 in SI.

**Figure 3:**
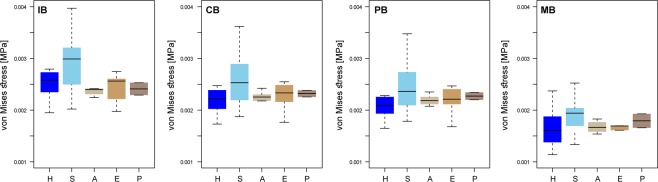
Box-plots of the percentile M50 values. Extant species are grouped by hardness of ingesta (H: Hard eaters; S: Soft eaters). IB: incisive bite; CB: canine bite; PB: premolar bite and MB: molar bite. For the fossil taxa, A: Australopithecus; P: Paranthropus; E: Early Homo. The median is the middle line of the box and whiskers represent the range.

Regarding the food hardness categories, previous results^32^ have clearly indicated that extant hard-food eaters have stiffer mandibles as compared to soft-food consumers (Fig. 3 when comparing the percentile M50 in all the models). The PERMANOVA test showed that in most cases mandibular stiffness can distinguish according to diet and food hardness categories (Table S8 in SI).

*Australopithecus* (*A. afarensis, A. africanus, A. sediba*) and *Paranthropus* (*P. robustus, P. boisei)* exhibit similar results for all the computed values, displaying values that would locate them within the range of extant primates that consume habitually hard-food items for the incisive bite. Nonetheless, the values of stress in the other configurations cannot be easily interpreted because they can be classified into both categories (i.e., hard vs. soft). *H. erectus* broadly showed a similar range of stresses as *Australopithecus* and *Paranthropus* but with weak results for their mandibles. It is worth to mention that *Paranthropus* present higher values of stress compared to the other fossils during molar bite. Figure S3 in SI correspond to boxplots of the percentiles 75^th^ and 95^th^. It is interesting that for the maximum von Mises stress values observed in the mandibles –which are those responsible of a hypothetical failure of the mandible- show a similar trend for both diet categories and food hardness.

### Geometric morphometrics

**Figure 4:**
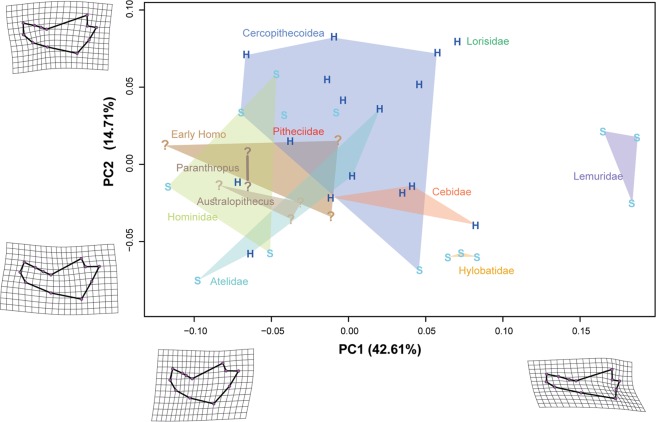
PCA of the mandibular shape variables (only the two first PCs are shown) including both the extant and fossil samples. The mean shape was warped to represent the variation along the two-plotted PC axes using the thin-plate spline method. H: Hard food eater; S: Soft food eater; ?: Fossil.

The PCA of the shape variables displays the morphological differences between the analysed specimens (Fig. 4). The first two PCs accounted 57.32% of the total shape variation, hence providing a fair approximation of the total amount of shape variation. PC1 distinguishes between relatively shorter and more ‘robust’ mandibles (e.g. most hominoids) as compared to those that are relatively elongated and slenderer (e.g. Lemuridae). The deformation grids display marked changes related to the position of the gonial angle and coronoid. On the other hand, PC2 mostly distinguishes some platyrrhines (e.g. the Atelidae) and some hominoids (e.g. the Hylobatidae) from the Cercopithecinae. The shape changes associated with this axis are related to differences in corpus thickness, posterior molar area, and gonial angle. As observed in Fig. 4 there is no clear trend distinguishing between hard and soft food eaters at least when observing the first two PCs. A significant phylogenetic signal was found for mandibular shape (K-mult: 0.3068; p-value: 0.001). The biomechanical and the shape data showed a moderate and significant covariation when accounting for phylogenetic relatedness (r-PLS: 0.635; p-value: 0.0032; 9,999 permutations).

### Fossil classification

The best SVM model for the biomechanical data was obtained when cost = 2.4 (Average accuracy: 0.87; Average Cohen’s Kappa: 0.73), whereas for the morphometric dataset the best model was obtained when cost = 2.9 (Average accuracy: 0.83; Average Cohen’s Kappa: 0.66). A Cohen’s Kappa value of ~0.5 represents a decent agreement^53^; hence we were confident to use the obtained models to classify the fossil sample (Table 3). Both the biomechanical data as well as the shape PCs classified almost all fossils as soft-eaters.

**Table 3:** Prediction results for the fossil sample using the SVM models for both the Biomechanical data from FEA and the Morphometric data obtained using GM.

Species/Specimen	Biomechanical data		Morphometric data	
	Posterior probabilities		Posterior probabilities	
	Hard-food eater	Soft-food eater	Hard-food eater	Soft-food eater
*Au. afarensis*	0.324	0.68	0.17	0.83
*Au. africanus*	0.32	0.68	0.11	0.89
*Au. sediba*	0.25	0.75	0.17	0.83
Asian *H. erectus*	0.35	0.65	0.57	0.43
Georgian *H. erectus*	0.41	0.59	0.12	0.88
*H. rudolfensis*	0.26	0.74	0.14	0.86
*P. boisei*	0.09	0.91	0.17	0.83
*P. robustus*	0.42	0.58	0.27	0.73

## Discussion

It has been previously proposed that a species’ dietary niche should be reflected in its ecological functioning and thus its morphology^54^. In reverse, the science of functional morphology attempts to assess niche occupation using morphology. General assumptions relating diet and morphology of the mandible have been tested previously^32^ and suggest robust evidence in extant primates that hard-eaters have stiffer jaws when compared to primates that comminute softer ingesta^55,56^. From a biomechanical perspective, stiffer jaws exhibit larger areas of lower stress values along with lower peak values as compared to less robust jaws, which present more areas with higher stresses. Consequently, stiffer jaws can better withstand high biting forces, thus enabling them to crush harder items. Irrespective of whether a particular species relies on one kind of food or another, having a stronger and stiffer jaw provides the ability to cope with higher forces.

Inferring feeding adaptations in fossil taxa is not an easy task, and consequently reconstructions of hominin paleobiology have relied on various analytical approaches that have provided different or even contradictory results. All these approaches exhibit limitations, but each one of them contributes incrementally toward the understanding of feeding behaviour in hominins and its evolution. Testing the hypothesis that hard foods exercised a significant selective pressure that influenced the evolution of cranio-mandibular morphology has yielded inconsistent results.

On one hand, some biomechanical analyses seem to indicate that hard foods were an important component of hominin diets^2,11,16,28^. Nonetheless, this seems to be inconsistent with data derived from dental microwear, which tend to indicate a softer diet^3,10,24,25^ or habitat reconstruction based on stable isotopes^5,19,57^. It is likely that the ingesta of the early hominins probably included substantial components of both soft and hard elements, either on a regular basis or as a fallback option^3,20^. What is relevant to clarify is what ingesta related signal are we detecting from different sources of evidence to elucidate the relative contributions of these different dietary demands. Therefore, assessing this topic requires acknowledging that different analytical approaches (e.g. microwear/microtexture, biomechanics, morphology and enamel chip/crack frequencies) are in fact evaluating hypotheses that encapsulate a variety of aspects of diet, ingesta, potential ingesta and environmental conditions related to ingesta and their availability. Consequently, these different methodologies enable the reconstruction of different and partly complementary palaeobiological aspects that relate to the properties of diet among others. For example, microwear/microtexture and isotopic analyses are first choice tools when reconstructing general dietary patterns, although they infer highly complementary aspects of ingesta and temporally cover several magnitudes. Thus, they are quite restricted when testing adaptive hypothesis based on gross morphology. On the other hand, FEA enables the assessment of the mechanical foundations of form-function relationships but does not provide any specific information about the foods consumed.

In this context, this paper follows a broad-scale comparative approach that put into perspective the hard vs. soft ingesta discussion in the context of primate evolution. This means that we compared both the morphology and the biomechanical performance inferred from hominin fossils within a comparative framework of primates with known diets that allowed us to classify them as either hard or soft eaters. It is important to bear in mind that when performing dietary inferences using tools such as FEA or GM, it is a major category of biomechanically challenging ingesta that bears most on morphology rather than a specific component that even might constitute the essence of a diet. For instance, when stating that the diet of a taxon represented by specific fossils, was predominantly soft does not imply that this specimen was incapable of consuming harder items, but rather that harder elements were probably a minor component of its diet, which in turn seems to be reflected in its biomechanical performance and morphology. In addition, it is important to bear in mind that any classification scheme is rough a simplification of the actual feeding behaviour observed in the actual animals since discretization is always a complicated task. Nevertheless, we based our binary classification scheme on previous publications that discretized feeding behaviour based on the material properties of the main ingesta of the analysed species^58^.

As a general overview, FEA results in Fig. 3 would suggest food pre-processing using the frontal dentition. Their anterior bite position exhibited low stress values comparable to actual hard food eaters. This supports the inference that the mandibles of fossil hominins were functionally adapted to withstand the acting forces associated with crushing mechanically resistant foods. On the other side, the canine, premolar and molar bites results could also indicate grinding action on soft, but tough and compliant foods or the consumption of pre-processed items. These results could mean that the analysed individuals did not habitually feed on hard-foods but rather maintain the biomechanical requirements to do so, when relying on mechanically more challenging ingesta as a fallback option.

In the case of the *A. africanus* and *A. afarensis*, our data are consistent with Delezene *et al*.^10^. They suggest no evidence that hard objects were habitually processed with the premolars or consumed as a regular dietary source in these species. Additionally, microwear and morphological studies of *A. africanus* also support a softer diet^12^, even though biomechanical studies have shown that its facial skeleton was well adapted to withstand loads due to the ingestion of large, mechanically protected food objects which had to be cracked open in the first place with the premolars^11^. Nevertheless, *A. sediba*, was recently identified as a species not particularly adapted to produce high molar bite forces^13^, thus limiting its ability to comminute hard foods, even though dental microwear data have been previously interpreted as evidence of the opposite^14^.

The genus *Paranthropus* is characterized by robust crania and mandibles that led scholars to consider them either specialized on hard or tough foods^9,59^, or to at least occasionally consume such hard items^3,21^. The massive molars and robust jaw of this genus have been traditional understood as adaptive traits related to the usual mastication of hard nuts and seeds; hence the ‘Nutcracker man’ nickname given to *P. boisei*’s holotype OH5 at the time of its discovery. This particular suite of morphological traits observed in *Paranthropus* were interpreted as the completion of an evolutionary trend in hominin evolution for the adaptation to more open habitats during the Plio-Pleistocene^19^. Yet, when microwear or isotopic values are considered, contradictory inferences arise again. For example, it has been suggested that the toughness of the ingesta of *P. boisei* ranged within the values obtained for *A. africanus* and that they did not consume harder and more brittle foods than *P. robustus*^3^. Despite the demonstrated capacity of the skull of *Paranthropus* to cope with high bite forces^16^, the biomechanical inference from FEA models by Wroe *et al*.^8^ does not suggest large differences between the stresses observed in *A. africanus* and *P. boisei* models. For instance, the observed results in the FEA models herein supports the hypothesis of Ungar and Sponheimer^60^ that *Paranthropus* did not require a particularly robust mandible for a diet dominated by tough but not particularly hard food. Furthermore, the observed stress patterns are closer to those of *Theropithecus*, a species adapted to the consumption of tough grasses. This grass-based diet is further suggested by the C4 dominated isotopic signal found in *P. boisei*^19^.

The results presented here show a rather unexpected stress pattern for *Paranthropus* during molar bite. In this biting position, both *P. boisei and P. robustus* present the highest stress values in the hominin clade. This would suggest a surprisingly not resistant mandible, at least, for this loading case. Even though our results are inconsistent with earlier inferences suggesting that *P. boisei and P. robustus* were able to generate exceptionally high bite forces^2^, we consider that they are not generally contradictory with related niche concepts. The planar FEA models created in this work have the same average “thickness”, which proved a good approximation for the models created (i.e., extant and fossils) despite these taxa have a remarkably wide corpus in the alveolar section. The framework established in this work combining biomechanical and morphological data infers a high soft-food probability for the genus *Paranthropus*, which is in agreement with the most recent isotopic^19^ and microwear^3^ studies. The differences between *P. robustus* and *P. boisei* observed in the posterior probabilities obtained in our SVM classification are also consistent with the suggestion that both *Paranthropus* species had different dietary preferences, which is also supported by dental microwear and isotopic analysis. This could be an environmental signal since *P. boisei* inhabited the wooded and open grasslands of East Africa at roughly the same time *P. robustus* was occupying climatically and plant geographically largely different South Africa, hence probably encountering largely different resources.

Finally, our biomechanical data also supports previous results in microwear of early *Homo* that indicates a diet consisting neither of very fracture-resistant foods nor for very tough and brittle items^24,25,61^. Furthermore, only a few studies investigate the diet of *H. rudolfensis*, but even though tooth morphology suggests plant material and probably meat^61^, there is no convincing evidence of hard-food in the diet of these specimens.

Mandibular shape variables have been mostly interpreted at an interspecific level as the result of constraints imposed by phylogenetic history and the individuals masticatory function^62,63^. In fact it has been shown that primate mandibular morphology exhibits a noticeable phylogenetic signal, which is modified by adaptive constrains imposed by specific dietary regimes^63^. Despite the limitations of our sample (we could not consider intraspecific variation at a significant level, mainly because of the scarcity of the fossil record), the PCA of mandible shape clearly separated most of the major primate clades in the morphospace. Testing for hardness of the diet, however, does not produce a clear pattern. This suggests that phylogenetic heritage dominates over current dietary niche in shaping mandibular morphology. These findings are consistent with other published data based on larger samples^63^. Finally, we found a significant covariation between stress values and mandibular shape even when considering the phylogenetic structure of the data. This in turn suggests that dietary traits do shape mandibular morphology at least partially once accounted for phylogeny.

Combining both biomechanics (FEA) and morphology (GMM) thus can be interpreted that fossil individuals investigated here do not suggest adaptive traits towards hard-food consumption but likely relied on soft-foods (Table 3). Of course, this does not imply that harder food items were generally avoided, an argument also brought forward^3,20^. This notion is supported by most of the recent microwear and isotopic analyses that challenge the “hard foods” hypothesis previously seen supported based on “classical”^2^ and some quantitative approaches^11^.

If we consider that GMM and biomechanical performance do not indicate “hard food” adaptive traits in most of investigated hominin taxa, it is important to mention that this does not hold true for the morphometric data of one of the *H. erectus* individuals we studied. Dental evidence suggests a likely shift in diet in early *Homo*, most noticeable in *H. erectus*, as compared to their archaic hominin forebears. Broadened subsistence patterns and increased opportunistic foraging strategies will always mean increased inclusion of mechanically more challenging ingesta, which biomechanically equal the necessity to cope with a wider range of properties related to fracture formation and propagation^61^. Niche expansion would thus explain the classification of the Zhoukoudian *H. erectus* reconstruction by Tattersall and Sawyer^39^ as a hard food eater, if not the reconstruction under consideration here was at the very robust end of (poorly established) variability in the sample. Support for this notion comes from Xing *et al*.^64^ who recently reported on the enamel-dentine junction (EDJ) of the Zhoukoudian Lower Cave hominin sample. Crenulation of the EDJ along with stout roots and the taurodontism observed suggests adaptive traits to withstand high biomechanical demands that partly may compensate functional losses due to dentognathic reduction. Supporting evidence also comes from Kundrát *et al*.^65^ who suggest biting of some solid objects which is consistent with comparatively demanding biomechanical requirements. However, further analyses are required in order to better understand this result, particularly when considering that the remainder of the *H. erectus* sample is classified as soft-food eaters, as well as when taking into account that the posterior probabilities obtained for both categories are quite similar.

## Supplementary information

Supplementary Information.
