# Supplementary material for: Broad-scale morpho-functional traits of the mandible suggest no hard food adaptation in the hominin lineage
Source: Sci Rep. 2020 Apr 22;10:6793. doi: 10.1038/s41598-020-63739-5 (PMC7176708; doi:10.1038/s41598-020-63739-5)
Supplement: Supplementary file 1 — Supplementary Information. [file 41598_2020_63739_MOESM1_ESM.pdf]

Broad-scale morpho-functional traits of the mandible suggest no hard food adaptation in the hominin lineage.

Jordi Marcé-Nogué[1,2], Thomas A. Püschel[3], Alexander Daasch[1] and Thomas M. Kaiser[1]

[1]*Centrum für Naturkunde, University of Hamburg, Martin-Luter-King-Platz 3 20146 Hamburg, Germany*

[2] *Institut Català de Paleontologia M. Crusafont, Universitat Autònoma de Barcelona, Cerdanyola del Vallès, Barcelona 08193, Spain*

[3]*Primate Models for Behavioural Evolution, Institute of Cognitive and Evolutionary Anthropology, University of Oxford, 64 Banbury Road, Oxford OX2 6PN, United Kingdom.*

**corresponding author: jordi.marce.nogue@icp.cat*

1. Case-specific fossil model reconstruction procedures

***Au. afarensis* 1** is based on the reconstruction made by Kimbel et al. [1], which comprises a composite skull and mandible. Both casts were available as part of the CeNak collection (Hamburg). Mandibular thickness was directly measured from the cast at the first premolar, the mid-point of the mesio-distal length of the premolar and molar series, and at the posterior end of the molar series, respectively the third molar.

***Au. afarensis* 2** was generated using the photographs from a research cast of A.L. 444-2 (cranium with mandible), housed at the Smithsonian Institution. The thickness of the model was derived from the corpus breadth measurements at the first molar as compiled by Skinner et al. [2].

***Au. afarensis* 3** model was produced using a lateral photograph of the original A.L. 822-1 individual (cranium with mandible) from Kimbel and Rak [3]. Mandibular thickness was derived from the corpus breadth measurements at the level of the first molar by Villmoare et al. [4].

***Au. africanus* 1** is represented by the cranium Sts 71 and the mandible Sts 36, likely to represent a male individual. Both specimens were available as virtual. stl models created using photogrammetry and based on research casts curated at the CeNak collection (Hamburg). In Sts 36 the upper part of the coronoid process is not preserved, and thus the area was reconstructed based on the morphology of Sts 52b, another mandible from Member 4 type site at Sterkfontein and also currently attributed to *Au. africanu*s. The thickness of the models positioned at P4 and M1 for both *Au. africanus* individuals were taken from breadth measurements of De Ruiter et al. [5].

***Au. africanus* 2** combines the cranium of Sts 5 and the mandible Sts 52b. A photogrammetry model was created based on a research cast available from the CeNak collection, while for the mandible Sts 52b the digital reconstruction from Benazzi et al. [6] was available thanks to the courtesy of Dr Stefano Benazzi. Thickness values are also from De Ruiter et al. [5].

Au. sediba is based on a series of original photographs taken by TMK of the skull remains (cranium UW88-50 and mandible UW88-8) of MH1 the male individual from Malapa cave

(South Africa). Thickness measurements of the mandible were inferred from breadth measurements [5] at P4 and M1 positions.

P. robustus is represented by the DNH 7 skull specimen from the Drimolen cave site (South Africa). The model was generated using the lateral photography of the original specimen provided by Keyser [7]. Parts of the upper ramus of the associated mandible are not preserved. Since the applied approach requires complete models, the missing section was reconstructed based on a photogrammetry derived .stl model from the likely female mandible SK 23 from Swartkrans cave, attributed to *P. robustus*, (CeNak collection of research casts). Thickness measurements at M1 and M2 of the DNH 7 mandible were taken from breadth measurements of Keyser [7].

P. boisei represents the composite of the OH 5 cranium from Olduwai Bed 1 and the Peninj 1 mandible from Lake Natron. The virtual models of a research casts (CeNak collection) were combined. As the left side of Peninj 1 lacks the articular and coronoid process, the ramus was digitally mirrored, so that only a small part of the coronoid process of the restored ramus had to be reconstructed. The thickness of the model was derived from the corpus breadth measurements at the first molar as compiled by Skinner et al. [2].

In the case of the ***H. rudolfensis*** skull, a photogrammetry-based model of a research cast (CeNak digital collection) of KNM-ER 1470 from East Turkana (Kenya) was combined with a CT-based reconstruction of the KNM-ER 60000 mandible [8]. Since the zygomatic arch of KNM-ER 1470 is missing (excluding some small portions), the origin of the masseter was placed in between. Measurements of KNM-ER 60000 are from Leakey et al. [8] at the level of P4, M1 and M2.

Two specimens of early Homo attributed to *H. erectus* from Dmanisi (Georgia) were analyzed. For **Georgian *H. erectus* 2** the lateral aspect of the cranium D2700 based on a photography by Vekua (2002) was used. The skull was mirrored to align with the photograph of mandible of D2735 [1011], as the left side of the mandible is more complete. The zygomatic arches of D2700 are not preserved, however this did not hinder the reconstruction of the origin area of the masseter muscle complex. Thickness values of the mandibular corpus at P4 and M2 were taken from Van Arsdale and Lordkipanidze [12].

Georgian *H. erectus* 5 is based on a lateral photograph of the original D4500 cranium [11] was paired with an image of the mandible D2600 [13]. The specimens thus represents "skull 5" from Dmanisi. This specimen [11] seems to be the most distinct of the Dmanisi hominin sample. Since the mandible lacks a large part of its angle and a smaller part from the coronoid process, we decided to digitally reconstruct those areas. The thickness of the model was derived from the corpus breadth measurements at the first molar as compiled by Skinner et al. [2].

The **Asian *H. erectus*** is a generalized reconstruction of the male "Peking Man" skull from the Zhoukoudian lower cave stratum (China) by Tattersall and Sawyer [14]. It was available as a cast (cranium with mandible) in the CeNak collection (Hamburg). Thickness values for the model were measured on the cast at the first premolar, the mid-point of the mesio-distal length of the premolar and molar series, and at the posterior end of the molar series, respectively the third molar.

2. Further Details of the Support Vector Machine algorithm

Two support vector machine (SVM) models were trained using the extant data (i.e. both biomechanical and morphometric) and then used to classify the fossil sample. SVMs correspond to a group of related learning methods for classification and regression, which are considered to be among the most powerful and flexible modelling techniques [15]. In fact, SVMs are considered one of the most successfully applied techniques for pattern recognition [16]. SVMs are based on machine learning theory, which is traditionally concerned with balancing empirical risk and the capacity of a learning machine in order to obtain a small actual risk (i.e., a good generalization performance) [17,18]. SVMs are part of a wider group of techniques known as kernel methods that owe their name to the use of kernel functions [19]. These functions enable these algorithms to work in a high-dimensional space without estimating the coordinates of the data in that space, but rather by just computing the inner products between points in a suitable feature space, which reduces computational cost even in very high-dimensional spaces [19]. Different SVM algorithms use different types of kernel functions, such as linear, radial, polynomial, among other possible kernels [20].

When the task involves linearly separable binary classification, SVM considers the input data as two sets (i.e., two-class) of p-dimensional vectors (i.e., data point with p features), and classification decisions are based on linear combinations of the variables. An SVM classifier will try to generate a separating p–1 dimensional hyperplane in that space, which maximizes the separation between the two sets of data [21]. The larger the distance of the hyperplane to the neighboring data points of both classes, the better the generalization is, and, consequently, the smaller the empirical classification error obtained by the classifier will be. In certain cases, the categories are not linearly separable or the separation of more than two sets of data is needed. The first problem can be solved by using kernels that project the input data to a high-dimensional or even infinitely dimensional variable space [21]. Expansion of SVMs from two-class (binary) to multiclass problems can be performed by decomposing a single multiclass problem into several binary problems. In the present case, we applied an SVM algorithm using a linear kernel to binary classification problem (soft vs. hard food categories).

Our models were generated using the 'caret' package for R [22]. The two SVM models used a linear kernel. The 'caret' package provides a grid search where it is possible to specify tuning parameters for the models, which in our case corresponded of 'cost' values. We first started with an automatic grid search using the 'tuneLength' option to indicate the number of different values to try for each algorithm parameter (we initially set this parameter to 10). This only supports integer algorithm parameters, thus providing a quick first guess as to what values to try and which models are more promising. The most accurate model was further tuned by setting a manual grid search that specified a range of 'cost' values closer to the one used in the best model obtained by the automatic grid search. Then using the best model, the paleoanthropological fossil sample was classified into the different food hardness categories.

We computed the overall classification accuracy in order to measure the performance of the classification models. Additionally, Cohen's Kappa was also calculated. This statistic can range between −1 and 1, where a value of 0 means that there is no concordance between the observed and predicted classes, whilst a value of 1 would indicate perfect agreement of the model prediction and the observed classes. Negative values are indicative that the

prediction is in the opposite direction of the truth, but large negative values are rare when dealing with predictive models [23]. The abovementioned performance values were computed using a leave-one-subject-out cross validation criterion.

3. Tables and Figures

Table S1 - FEA characteristics of the models: jaw thickness. The thickness of the model was assumed to be constant throughout the mandible and this value was obtained from the individual average of these three measurements: THK1, THK2 and THK3

	Mesh Elements	THK1 [mm]	THK2 [mm]	THK3 [mm]	Average [mm]
**EXTANT MODELS**					
Alouatta seniculus	95191	8.37	7.76	7.05	7.73
Aotus trivergatus	83666	3.48	3.12	3.24	3.28
Ateles geoffroyi	133162	5.49	5.28	5.09	5.29
Brachyteles arachnoides	104825	7.31	7.40	9.33	8.01
Callithrix jacchus	74483	2.61	2.10	2.17	2.29
Cebus apella	96382	7.60	6.63	8.51	7.58
Cebus capucinus	82314	6.28	5.44	6.36	6.03
Cercocebus torquatus	209838	6.31	5.07	5.46	5.61
Chlorocebus aethiops	74546	5.97	5.85	7.04	6.29
Eulemur fulvus	59185	4.36	4.00	3.64	4.00
Gorilla gorilla	70064	22.13	18.16	24.78	21.69
Hapalemur griseus	79717	4.62	4.34	4.54	4.50
Homo sapiens	70836	16.23	15.52	14.32	15.36
Hylobates lar	89248	8.16	6.08	7.92	7.39
Hylobates moloch	90576	7.79	6.80	7.15	7.25
Hylobates muelleri	90225	7.17	5.61	5.66	6.15
Lemur catta	82229	3.63	3.52	3.64	3.60
Macaca fascicularis	78363	15.34	9.93	11.15	12.14
Macaca fuscata	72410	12.30	10.33	10.28	10.97
Macaca mulatta	80346	10.20	8.08	9.36	9.21
Macaca nemestrina	83043	11.68	9.95	8.72	10.12
Nycticebus coucang	80034	4.72	4.07	4.06	4.28
Pan troglodytes	67537	16.25	13.51	16.23	15.33
Papio cynocephalus	102591	14.07	12.21	13.07	13.12
Papio ursinus	80268	13.84	11.55	11.64	12.34
Pithecia pithecia	84555	5.13	4.64	4.60	4.79
Pongo pygmaeus	73035	17.47	13.03	15.14	15.21
Saimiri sciureus	75186	3.55	2.80	3.60	3.32
Therophitecus gelada	184535	11.36	10.74	12.31	11.47
Trachypithecus cristatus	96252	7.10	6.05	6.20	6.45
**FOSSIL MODELS**					
Australopithecus afarensis 1	91182	17.00	18.00	20.00	18.33
Australopithecus afarensis 2	97219	-	23.00	-	23.00
Australopithecus afarensis 3	93138	-	18.00	-	18.00
Australopithecus africanus 1	83041	-	19.00	20.00	19.50
Australopithecus africanus 2	81464	-	21.50	24.50	23.00
Australopithecus sediba	81465	-	17.60	18.20	17.90
Paranthropus robustus	78541	-	20.90	29.10	25.00
Paranthropus boisei	76117	-	-	29.00	29.00
Homo rudolfensis	80286	21.00	20.00	21.00	20.67
Georgian *Homo erectus 2*	86962	19.4	22.7	-	21.05
Georgian *Homo erectus 5*	85054	21.9	21.8	-	21.85
Asian *Homo erectus*	82996	12.00	15.50	18.00	15.17

Table S2 – FEA characteristics of the models: Calculated surfaces for the masseter, temporalis and pterygoid in both jaw and skull and calculated surface of the model.

	Jaw Masseter Surface [mm^2]	Jaw Temporalis Surface [mm^2]	Jaw Pterygoid Surface [mm^]	Skull Masseter Surface [mm^2]	Skull Temporalis Surface [mm^2]	Skull Pterygoid Surface [mm^]	Model Surface [mm^2]
**EXTANT MODELS**							
Alouatta seniculus	1210.20	39.72	559.79	95.38	1548.20	48.64	2134.10
Aotus trivergatus	229.49	29.38	143.19	20.80	374.72	5.47	452.80
Ateles geoffroyi	611.83	57.83	299.92	99.66	1942.59	26.94	1342.21
Brachyteles arachnoides	1387.70	104.06	868.11	130.36	2022.10	36.98	2303.00
Callithrix jacchus	100.48	9.42	57.51	12.93	351.84	5.28	186.10
Cebus apella	408.01	31.00	227.85	40.96	1747.40	24.22	957.80
Cebus capucinus	292.14	30.97	153.66	50.39	1725.60	25.99	784.32
Cercocebus torquatus	518.02	41.06	252.98	62.40	1736.40	31.72	1198.60
Chlorocebus aethiops	276.50	21.73	104.39	38.87	1270.10	10.82	741.84
Eulemur fulvus	211.10	62.93	88.06	53.91	683.53	24.42	590.76
Gorilla gorilla	5468.40	649.75	2179.90	1154.50	9953.30	439.31	11041.00
Hapalemur griseus	206.84	38.64	109.41	42.67	460.60	13.91	449.38
Homo sapiens	1480.20	231.49	853.38	250.49	13453.00	98.46	4440.90
Hylobates lar	333.94	43.05	128.97	96.10	1530.00	11.57	911.05
Hylobates moloch	291.63	46.59	140.69	36.55	2002.20	15.55	895.21
Hylobates muelleri	367.45	28.43	165.76	76.55	2051.10	32.99	893.75
Lemur catta	181.98	16.71	60.20	25.02	568.65	9.31	460.63
Macaca fascicularis	1202.20	95.75	540.96	174.80	2494.10	78.74	3130.20
Macaca fuscata	935.63	96.06	482.53	112.64	1678.60	52.75	2178.20
Macaca mulatta	718.36	91.05	297.19	84.36	2263.30	31.44	1762.80
Macaca nemestrina	1065.90	140.60	441.97	139.01	2185.00	80.14	2655.60
Nycticebus coucang	204.35	22.42	62.17	25.16	411.24	7.39	445.52
Pan troglodytes	1655.10	148.52	1105.60	298.02	4321.60	80.27	4179.40
Papio cynocephalus	1716.70	202.75	809.13	242.97	4076.10	120.47	4096.60
Papio ursinus	1983.80	248.96	859.08	290.36	3510.00	131.23	5011.00
Pithecia pithecia	420.41	35.74	260.62	51.95	1140.40	22.22	831.37
Pongo pygmaeus	1907.60	182.40	868.42	278.59	5945.80	103.02	4552.50
Saimiri sciureus	111.61	14.95	45.36	15.60	584.33	2.28	393.25
Therophitecus gelada	1758.10	85.81	864.91	287.94	3732.90	69.10	4115.10
Trachypithecus cristatus	195.98	42.70	414.13	64.77	1417.00	26.55	952.96
**FOSSIL MODELS**							
Australopithecus afarensis 1	3132.29	320.05	1453.56	400.49	4289.36	128.84	8106.67
Australopithecus afarensis 2	4173.57	394.81	1649.22	940.10	7276.95	276.41	8716.00
Australopithecus afarensis 3	3063.97	273.26	1245.43	403.28	5143.92	138.06	5747.76
Australopithecus africanus 1	2154.22	289.88	963.93	352.93	6469.13	112.00	5146.34
Australopithecus africanus 2	2086.40	194.01	821.53	325.93	5264.00	86.32	4248.54
Australopithecus sediba	2095.97	207.13	829.36	381.64	5059.74	172.38	4300.12
Paranthropus robustus	2403.85	339.01	1182.67	405.13	4825.85	77.58	4875.92
Paranthropus boisei	3442.87	412.31	1109.91	955.43	8470.69	223.52	6824.52
Homo rudolfensis	2150.13	266.93	873.46	486.25	7725.78	95.09	4251.78
Georgian *Homo erectus 2*	1882.39	240.35	764.61	488.63	7895.12	103.77	4599.49
Georgian *Homo erectus 5*	2914.03	335.12	1512.60	439.18	7084.29	150.05	6705.37
Asian *Homo erectus*	1680.60	147.49	784.93	252.91	6499.35	113.63	4437.86

Table S3 – FEA characteristics of the models: Total muscle force and muscular forces for each model.

	Total Muscle Force [N]	Masseter force [N]	Temporalis force [N]	Pterygoid force [N]
**EXTANT MODELS**				
Alouatta seniculus	0.3488	0.1300	0.1582	0.0606
Aotus trivergatus	0.0682	0.0213	0.0343	0.0126
Ateles geoffroyi	0.1893	0.0443	0.1246	0.0204
Brachyteles arachnoides	0.3758	0.1254	0.1756	0.0748
Callithrix jacchus	0.0306	0.0065	0.0205	0.0036
Cebus apella	0.2292	0.0415	0.1644	0.0233
Cebus capucinus	0.1649	0.0248	0.1271	0.0130
Cercocebus torquatus	0.1899	0.0417	0.1277	0.0205
Chlorocebus aethiops	0.1673	0.0306	0.1255	0.0112
Eulemur fulvus	0.0950	0.0224	0.0631	0.0095
Gorilla gorilla	2.2270	0.7432	1.1899	0.2939
Hapalemur griseus	0.0932	0.0267	0.0534	0.0132
Homo sapiens	1.0000	0.1057	0.8361	0.0582
Hylobates lar	0.2179	0.0437	0.1599	0.0143
Hylobates moloch	0.2119	0.0274	0.1714	0.0131
Hylobates muelleri	0.1796	0.0293	0.1372	0.0131
Lemur catta	0.0754	0.0181	0.0512	0.0061
Macaca fascicularis	0.6637	0.1993	0.3748	0.0897
Macaca fuscata	0.5003	0.1562	0.2644	0.0797
Macaca mulatta	0.3780	0.0870	0.2553	0.0356
Macaca nemestrina	0.5094	0.1515	0.2923	0.0656
Nycticebus coucang	0.0883	0.0277	0.0523	0.0084
Pan troglodytes	0.9684	0.2486	0.5689	0.1509
Papio cynocephalus	0.8203	0.2243	0.4897	0.1064
Papio ursinus	0.8538	0.2765	0.4570	0.1204
Pithecia pithecia	0.1350	0.0330	0.0822	0.0198
Pongo pygmaeus	1.0030	0.2361	0.6619	0.1049
Saimiri sciureus	0.0643	0.0106	0.0498	0.0040
Therophitecus gelada	0.7190	0.2164	0.4038	0.0988
Trachypithecus cristatus	0.1946	0.0235	0.1314	0.0397
**FOSSIL MODELS**				
Australopithecus afarensis 1	1.6129	0.5860	0.7645	0.2625
Australopithecus afarensis 2	2.0982	0.7293	1.0942	0.2746
Australopithecus afarensis 3	1.3335	0.4503	0.7035	0.1797
Australopithecus africanus 1	1.3669	0.3314	0.8933	0.1422
Australopithecus africanus 2	1.4649	0.4026	0.9108	0.1515
Australopithecus sediba	1.1470	0.3249	0.6907	0.1314
Paranthropus robustus	1.7058	0.5189	0.9541	0.2328
Paranthropus boisei	2.3410	0.7045	1.4229	0.2136
Homo rudolfensis	1.3168	0.2993	0.9075	0.1100
Georgian *Homo erectus 2*	1.3950	0.2908	0.9977	0.1065
Georgian *Homo erectus 5*	1.7483	0.4714	1.0431	0.2338
Asian *Homo erectus*	0.9873	0.2014	0.6923	0.0936

Table S4 – Percentile values of von Mises Stress for IB (Incisive Bite) and error percentages of the respective QIMs. In order to ensure a QIM, we computed the required errors to be sure that they fulfil the requirements described in [24].

	PEofAM (%)	PEofM (%)	M25 [MPa]	M50 [MPa]	M75 [MPa]	M95 [MPa]
**EXTANT MODELS**						
Alouatta seniculus	0.05	0.22	0.001578	0.002020	0.003061	0.005266
Aotus trivergatus	0.27	1.26	0.001235	0.001949	0.002932	0.005149
Ateles geoffroyi	0.11	1.12	0.001641	0.002402	0.003442	0.006040
Brachyteles arachnoides	0.81	2.87	0.001520	0.002006	0.003035	0.006004
Callithrix jacchus	1.05	2.04	0.001621	0.002675	0.004544	0.009055
Cebus apella	0.59	3.06	0.001516	0.002348	0.003326	0.005920
Cebus capucinus	0.74	3.47	0.001740	0.002552	0.003687	0.006658
Cercocebus torquatus	0.14	0.79	0.001692	0.002470	0.003359	0.005987
Chlorocebus aethiops	0.27	3.67	0.001671	0.002534	0.003635	0.007313
Eulemur fulvus	0.45	1.03	0.001902	0.003250	0.005669	0.012004
Gorilla gorilla	0.04	2.58	0.002198	0.003076	0.004193	0.007833
Hapalemur griseus	0.34	0.12	0.001438	0.002456	0.003842	0.008504
Homo sapiens	0.92	0.91	0.001483	0.002207	0.003163	0.005591
Hylobates lar	1.67	1.47	0.002117	0.003166	0.004778	0.009118
Hylobates moloch	0.17	1.07	0.001546	0.002389	0.003595	0.006678
Hylobates muelleri	0.13	0.18	0.002373	0.003485	0.005579	0.011988
Lemur catta	0.02	0.72	0.002295	0.003714	0.006359	0.013066
Macaca fascicularis	0.59	1.03	0.001926	0.002852	0.004033	0.006434
Macaca fuscata	0.48	1.63	0.001668	0.002723	0.003591	0.005783
Macaca mulatta	0.33	3.64	0.002249	0.003620	0.005023	0.008749
Macaca nemestrina	0.06	2.68	0.001679	0.002792	0.003773	0.006577
Nycticebus coucang	0.66	2.01	0.001471	0.002344	0.003277	0.006585
Pan troglodytes	0.50	0.02	0.002068	0.002989	0.004154	0.007226
Papio cynocephalus	0.04	1.56	0.001851	0.002605	0.003568	0.007141
Papio ursinus	0.47	0.47	0.001715	0.002687	0.003920	0.006742
Pithecia pithecia	0.19	0.43	0.001413	0.002068	0.003081	0.005379
Pongo pygmaeus	0.06	2.40	0.002574	0.003636	0.004938	0.009563
Saimiri sciureus	0.50	0.63	0.001850	0.002738	0.003917	0.006884
Therophitecus gelada	0.27	0.50	0.001803	0.002686	0.004077	0.006601
Trachypithecus cristatus	0.17	0.97	0.001965	0.002996	0.004337	0.007069
**FOSSIL MODELS**						
Australopithecus afarensis 1	0.22	3.27	0.001964	0.002646	0.003583	0.006531
Australopithecus afarensis 2	0.21	2.21	0.001761	0.002413	0.003285	0.005930
Australopithecus afarensis 3	0.28	2.69	0.001753	0.002241	0.002913	0.005357
Australopithecus africanus 1	0.22	0.13	0.001827	0.002417	0.003185	0.005630
Australopithecus africanus 2	0.23	1.35	0.001741	0.002307	0.003037	0.005427
Australopithecus sediba	0.18	0.19	0.001793	0.002408	0.003303	0.006011
Paranthropus robustus	0.04	2.51	0.001872	0.002531	0.003358	0.006221
Paranthropus boises	0.37	0.24	0.001717	0.002290	0.003090	0.005234
Homo rudolfensis	0.50	1.62	0.001634	0.002214	0.002807	0.004869
Georgian *Homo erectus 2*	0.13	0.91	0.001862	0.002746	0.003840	0.007085
Georgian *Homo erectus 5*	0.37	0.72	0.001909	0.002605	0.003512	0.006721
Asian *Homo erectus*	0.17	2.19	0.001395	0.001974	0.002666	0.004696

Table S5 – Percentile values of von Mises Stress for CB (Canine Bite) and error percentages of the respective QIMs. In order to ensure a QIM, we computed the required errors to be sure that they fulfil the requirements described in [24].

	PEofAM (%)	PEofM (%)	M25 [MPa]	M50 [MPa]	M75 [MPa]	M95 [MPa]
**EXTANT MODELS**						
Alouatta seniculus	0.16	0.22	0.001511	0.001874	0.002664	0.004596
Aotus trivergatus	0.63	0.47	0.001158	0.001779	0.002515	0.004552
Ateles geoffroyi	0.00	0.27	0.001486	0.002073	0.002887	0.005038
Brachyteles arachnoides	1.33	3.71	0.001024	0.001371	0.001858	0.003682
Callithrix jacchus	1.02	1.01	0.001409	0.002409	0.003564	0.007587
Cebus apella	0.53	1.49	0.001336	0.001973	0.002851	0.005133
Cebus capucinus	1.23	0.84	0.001440	0.002093	0.003121	0.005662
Cercocebus torquatus	0.22	0.55	0.001548	0.002126	0.002895	0.005284
Chlorocebus aethiops	0.31	2.35	0.001301	0.002182	0.003223	0.006587
Eulemur fulvus	0.49	1.18	0.001887	0.003133	0.005496	0.011770
Gorilla gorilla	0.09	1.89	0.002068	0.002768	0.003770	0.007056
Hapalemur griseus	0.35	0.10	0.001437	0.002432	0.003789	0.008400
Homo sapiens	1.07	0.25	0.001388	0.002065	0.002981	0.005314
Hylobates lar	1.92	1.08	0.001826	0.002777	0.004249	0.008329
Hylobates moloch	0.09	1.05	0.001234	0.002040	0.003201	0.006067
Hylobates muelleri	0.13	0.31	0.001940	0.003005	0.004824	0.010668
Lemur catta	0.02	0.41	0.002242	0.003617	0.006225	0.012854
Macaca fascicularis	0.44	1.13	0.001726	0.002417	0.003643	0.005746
Macaca fuscata	0.63	1.29	0.001570	0.002366	0.003250	0.005351
Macaca mulatta	0.40	0.20	0.002107	0.003049	0.004362	0.007744
Macaca nemestrina	0.17	1.15	0.001539	0.002472	0.003338	0.005663
Nycticebus coucang	0.80	1.16	0.001287	0.002146	0.002936	0.005868
Pan troglodytes	0.64	0.02	0.001826	0.002552	0.003585	0.006420
Papio cynocephalus	0.09	3.44	0.001581	0.002311	0.003154	0.006339
Papio ursinus	0.39	0.13	0.001439	0.002204	0.003620	0.006124
Pithecia pithecia	0.32	0.18	0.001277	0.001729	0.002415	0.004558
Pongo pygmaeus	0.07	2.95	0.002223	0.003095	0.004244	0.008622
Saimiri sciureus	0.36	0.16	0.001612	0.002297	0.003350	0.006110
Therophitecus gelada	0.07	0.20	0.001553	0.002342	0.003827	0.006068
Trachypithecus cristatus	0.45	0.46	0.001766	0.002531	0.003461	0.006193
**FOSSIL MODELS**						
Australopithecus afarensis 1	0.35	2.37	0.001737	0.002422	0.003296	0.006009
Australopithecus afarensis 2	0.32	1.80	0.001716	0.002283	0.003135	0.005743
Australopithecus afarensis 3	0.33	2.41	0.001730	0.002177	0.002785	0.004935
Australopithecus africanus 1	0.32	0.47	0.001728	0.002222	0.002951	0.005296
Australopithecus africanus 2	0.33	0.96	0.001668	0.002182	0.002871	0.005132
Australopithecus sediba	0.15	0.71	0.001667	0.002301	0.003139	0.005730
Paranthropus robustus	0.04	2.50	0.001872	0.002531	0.003358	0.006221
Paranthropus boises	0.37	0.24	0.001717	0.002290	0.003090	0.005234
Homo rudolfensis	0.60	1.49	0.001614	0.002159	0.002743	0.004651
Georgian *Homo erectus 2*	0.20	1.10	0.001718	0.002546	0.003577	0.006683
Georgian *Homo erectus 5*	0.44	1.52	0.001791	0.002484	0.003348	0.006440
Asian *Homo erectus*	0.07	1.95	0.001283	0.001764	0.002387	0.004344

Table S6 – Percentile values of von Mises Stress for PB (Premolar Bite) and error percentages of the respective QIMs. In order to ensure a QIM, we computed the required errors to be sure that they fulfil the requirements described in [24]

	PEofAM (%)	PEofM (%)	M25 [MPa]	M50 [MPa]	M75 [MPa]	M95 [MPa]
**EXTANT MODELS**						
Alouatta seniculus	0.27	1.12	0.001431	0.001784	0.002528	0.004424
Aotus trivergatus	0.82	1.37	0.001076	0.001702	0.002364	0.004306
Ateles geoffroyi	0.00	1.32	0.001354	0.001967	0.002740	0.004730
Brachyteles arachnoides	0.96	3.44	0.001437	0.001896	0.002595	0.004913
Callithrix jacchus	0.87	1.29	0.001248	0.002281	0.003271	0.006951
Cebus apella	0.60	1.04	0.001146	0.001893	0.002639	0.004769
Cebus capucinus	1.57	0.83	0.001276	0.002003	0.002896	0.005232
Cercocebus torquatus	0.25	0.36	0.001463	0.002028	0.002774	0.005104
Chlorocebus aethiops	0.34	1.60	0.001193	0.002079	0.003086	0.006327
Eulemur fulvus	0.47	0.58	0.001810	0.002980	0.005242	0.011331
Gorilla gorilla	0.22	1.64	0.002036	0.002656	0.003601	0.006791
Hapalemur griseus	0.36	0.44	0.001440	0.002360	0.003636	0.008155
Homo sapiens	0.92	0.87	0.001296	0.001984	0.002839	0.005105
Hylobates lar	0.09	0.18	0.001732	0.002522	0.003865	0.007803
Hylobates moloch	0.11	0.86	0.001169	0.001917	0.003038	0.005783
Hylobates muelleri	0.02	0.22	0.001698	0.002811	0.004478	0.009948
Lemur catta	0.04	0.68	0.002105	0.003475	0.005904	0.012340
Macaca fascicularis	0.34	1.17	0.001616	0.002282	0.003521	0.005586
Macaca fuscata	0.73	0.82	0.001529	0.002269	0.003150	0.005274
Macaca mulatta	0.40	1.01	0.001929	0.002866	0.004166	0.007411
Macaca nemestrina	0.15	0.15	0.001469	0.002204	0.003163	0.005449
Nycticebus coucang	0.89	0.57	0.001133	0.002012	0.002745	0.005241
Pan troglodytes	0.76	0.67	0.001742	0.002457	0.003431	0.006200
Papio cynocephalus	0.13	3.22	0.001484	0.002243	0.003078	0.006183
Papio ursinus	0.38	0.18	0.001358	0.002107	0.003521	0.005942
Pithecia pithecia	0.33	0.23	0.001191	0.001648	0.002254	0.004425
Pongo pygmaeus	0.12	2.28	0.002049	0.002890	0.003966	0.008148
Saimiri sciureus	0.16	0.68	0.001459	0.002144	0.003093	0.005718
Therophitecus gelada	0.04	0.38	0.001462	0.002231	0.003716	0.005921
Trachypithecus cristatus	0.36	0.44	0.001440	0.002360	0.003636	0.008155
**FOSSIL MODELS**						
Australopithecus afarensis 1	0.63	1.83	0.001528	0.002351	0.003101	0.005627
Australopithecus afarensis 2	0.45	1.85	0.001621	0.002200	0.003025	0.005607
Australopithecus afarensis 3	0.30	1.73	0.001698	0.002076	0.002628	0.004741
Australopithecus africanus 1	0.31	0.72	0.001678	0.002167	0.002874	0.005190
Australopithecus africanus 2	0.37	1.05	0.001582	0.002114	0.002763	0.004927
Australopithecus sediba	0.15	0.64	0.001600	0.002256	0.003044	0.005566
Paranthropus robustus	0.14	2.09	0.001735	0.002344	0.003075	0.005690
Paranthropus boises	0.37	0.24	0.001716	0.002290	0.003090	0.005234
Homo rudolfensis	0.64	1.07	0.001587	0.002113	0.002669	0.004556
Georgian *Homo erectus 2*	0.24	0.52	0.001570	0.002467	0.003433	0.006389
Georgian *Homo erectus 5*	0.44	1.54	0.001713	0.002405	0.003252	0.006253
Asian *Homo erectus*	0.04	1.27	0.001239	0.001680	0.002273	0.004183

Table S7 – Percentile values of von Mises Stress for MB (Molar Bite) and error percentages of the respective QIMs. In order to ensure a QIM, we computed the required errors to be sure that they fulfil the requirements described in [24]

	PEofAM (%)	PEofM (%)	M25 [MPa]	M50 [MPa]	M75 [MPa]	M95 [MPa]
**EXTANT MODELS**						
Alouatta seniculus	0.26	0.25	0.000998	0.001335	0.001940	0.003847
Aotus trivergatus	1.37	2.37	0.000573	0.001199	0.001732	0.003329
Ateles geoffroyi	0.14	3.68	0.000745	0.001482	0.001991	0.003630
Brachyteles arachnoides	1.33	3.71	0.001024	0.001371	0.001858	0.003682
Callithrix jacchus	0.50	0.79	0.000752	0.001632	0.002584	0.005030
Cebus apella	0.64	3.37	0.000316	0.001139	0.001819	0.003528
Cebus capucinus	1.33	1.60	0.000125	0.001383	0.002154	0.004053
Cercocebus torquatus	0.28	1.00	0.000739	0.001606	0.002143	0.003967
Chlorocebus aethiops	0.80	4.46	0.000069	0.001354	0.002325	0.004985
Eulemur fulvus	0.20	1.47	0.000486	0.001922	0.003106	0.006876
Gorilla gorilla	0.48	3.90	0.001278	0.002215	0.002867	0.005270
Hapalemur griseus	0.16	0.88	0.000559	0.001676	0.002435	0.004872
Homo sapiens	1.71	1.24	0.000454	0.001723	0.002355	0.004720
Hylobates lar	0.09	0.18	0.001732	0.002522	0.003865	0.007803
Hylobates moloch	0.09	2.75	0.000191	0.001474	0.002268	0.004532
Hylobates muelleri	0.23	1.11	0.000415	0.002033	0.003294	0.006973
Lemur catta	0.64	2.45	0.000678	0.002415	0.003623	0.007767
Macaca fascicularis	0.39	1.39	0.001015	0.001982	0.002851	0.004756
Macaca fuscata	1.01	1.53	0.001174	0.001790	0.002558	0.004728
Macaca mulatta	0.32	2.81	0.001135	0.002371	0.003263	0.005684
Macaca nemestrina	0.15	1.45	0.000761	0.001723	0.002398	0.004115
Nycticebus coucang	0.58	1.72	0.000779	0.001578	0.002265	0.003737
Pan troglodytes	1.07	3.36	0.000851	0.002045	0.002696	0.005170
Papio cynocephalus	0.20	3.99	0.000757	0.001846	0.002423	0.004585
Papio ursinus	0.54	1.21	0.000489	0.001711	0.002718	0.004581
Pithecia pithecia	0.28	0.53	0.000807	0.001170	0.001776	0.003785
Pongo pygmaeus	0.61	4.71	0.000700	0.002192	0.003078	0.006324
Saimiri sciureus	0.39	2.47	0.000294	0.001614	0.002340	0.004326
Therophitecus gelada	0.36	0.44	0.000768	0.001908	0.002963	0.005017
Trachypithecus cristatus	0.94	1.95	0.000839	0.001832	0.002434	0.004742
**FOSSIL MODELS**						
Australopithecus afarensis 1	1.15	3.78	0.000747	0.001827	0.002648	0.004500
Australopithecus afarensis 2	0.66	2.06	0.000858	0.001605	0.002398	0.004622
Australopithecus afarensis 3	0.56	4.93	0.001010	0.001538	0.002192	0.003648
Australopithecus africanus 1	0.72	4.58	0.001003	0.001765	0.002332	0.004315
Australopithecus africanus 2	0.68	5.58	0.000895	0.001582	0.002175	0.003759
Australopithecus sediba	0.35	4.66	0.000794	0.001723	0.002465	0.004186
Paranthropus robustus	0.04	2.50	0.001872	0.002531	0.003358	0.006221
Paranthropus boises	0.38	0.24	0.001717	0.002290	0.003090	0.005234
Homo rudolfensis	0.79	1.58	0.001093	0.001693	0.002326	0.003734
Georgian *Homo erectus 2*	0.40	0.87	0.000723	0.001697	0.002664	0.004565
Georgian *Homo erectus 5*	0.53	2.27	0.001121	0.002027	0.002879	0.005151
Asian *Homo erectus*	0.16	3.13	0.000775	0.001390	0.001840	0.003485

Table S8 - P-values for the Two-Way PERMANOVA for the four biting cases (IB: Incisive Bite, CB: Canine Bite, PB: Premolar Bite and MB: Molar Bite) when analyzing all the percentiles of Von Mises stress together. Significant values are in bold.

	**DIET**			**HARDNESS**
	**Omnivore vs. Frugivore**	**Omnivore vs. Folivore**	**Frugivore vs Folivore**	**Soft vs Hard**
**IB: Incisive Bite**	0.1274	0.4609	0.4617	0.0529
**CB: Canine Bite**	0.2424	0.2427	0.3763	0.0182
**PB: Premolar Bite**	0.0886	0.0898	0.4753	0.0079
**MB: Molar Bite**	0.0350	**0.0392**	0.5541	0.9954

Figure S1 - Phylogenetic tree and von Mises Stress distribution for each specimen under the four different bite cases: IB: incisive bite; CB: canine bite; PB: premolar bite and MB: molar bite. Phylogenetic tree from http://10ktrees.nunn-lab.org/.

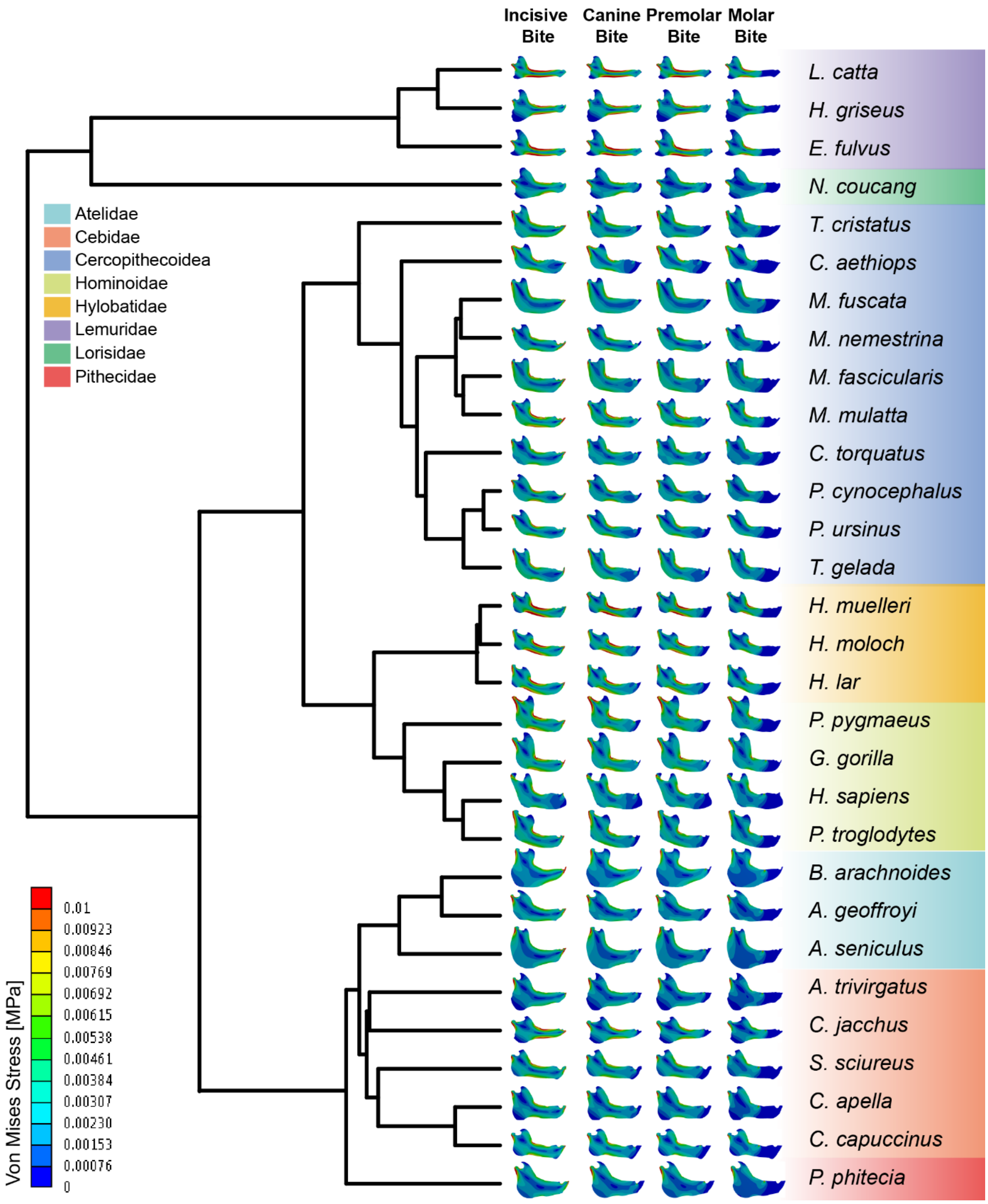

Figure S2 - Box-plots of Von Mises stress distributions when QIM is assumed for the actual primates and fossil hominins analyzed in the four biting cases. IB: incisive bite; CB: canine bite; PB: premolar bite and MB: molar bite

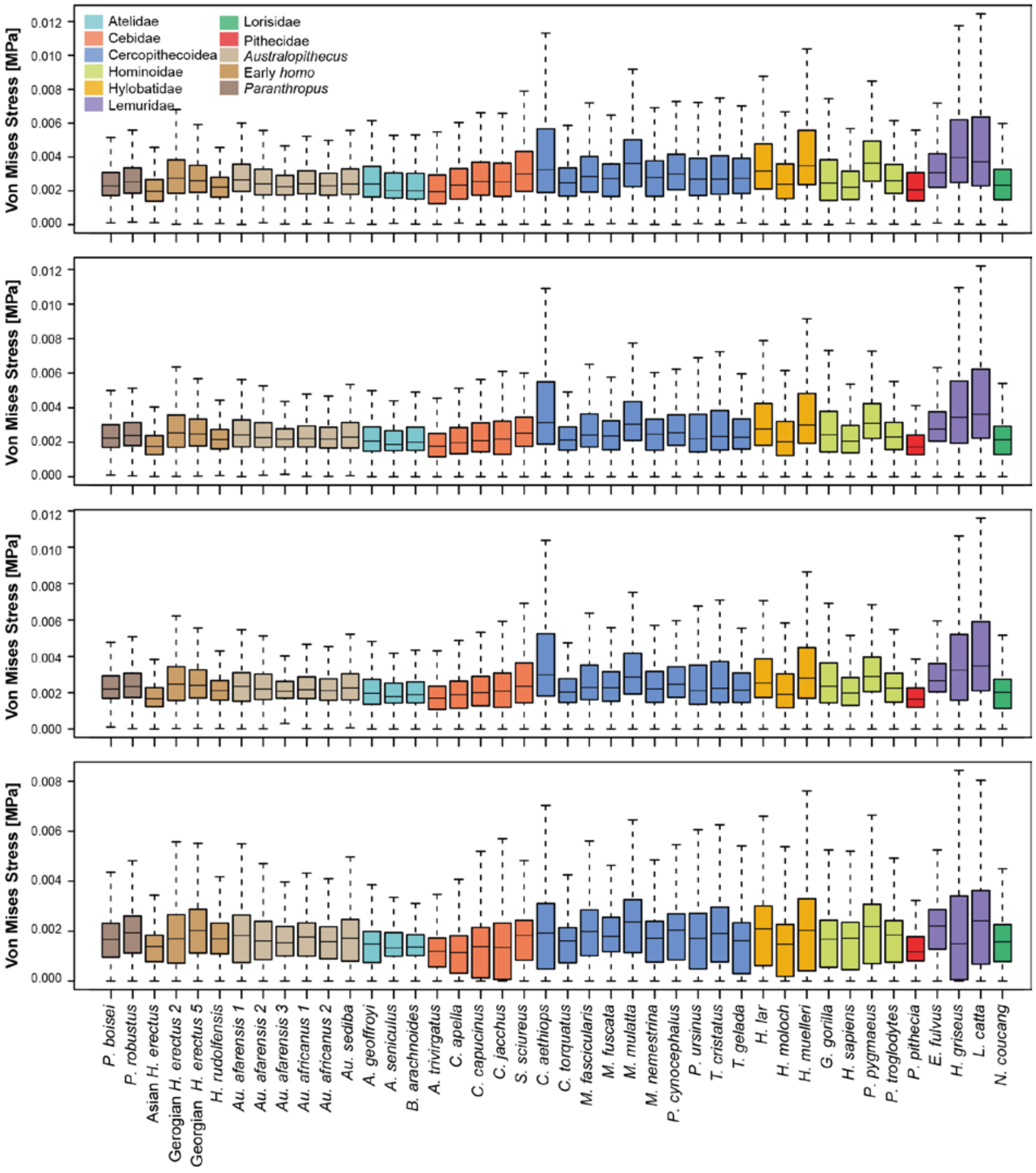

Figure S3 - Box-plots of the percentile M50, M75 and M95 values. Extant species are grouped by hardness of ingesta (H: hard eaters; S: soft eaters). IB: incisive bite; CB: canine bite; PB: premolar bite and MB: molar bite. For the fossil taxa, A: *Australopithecus*; P: *Paranthropus*; E: Early *Homo*. The median is the middle line of the box and the whiskers represent the range.

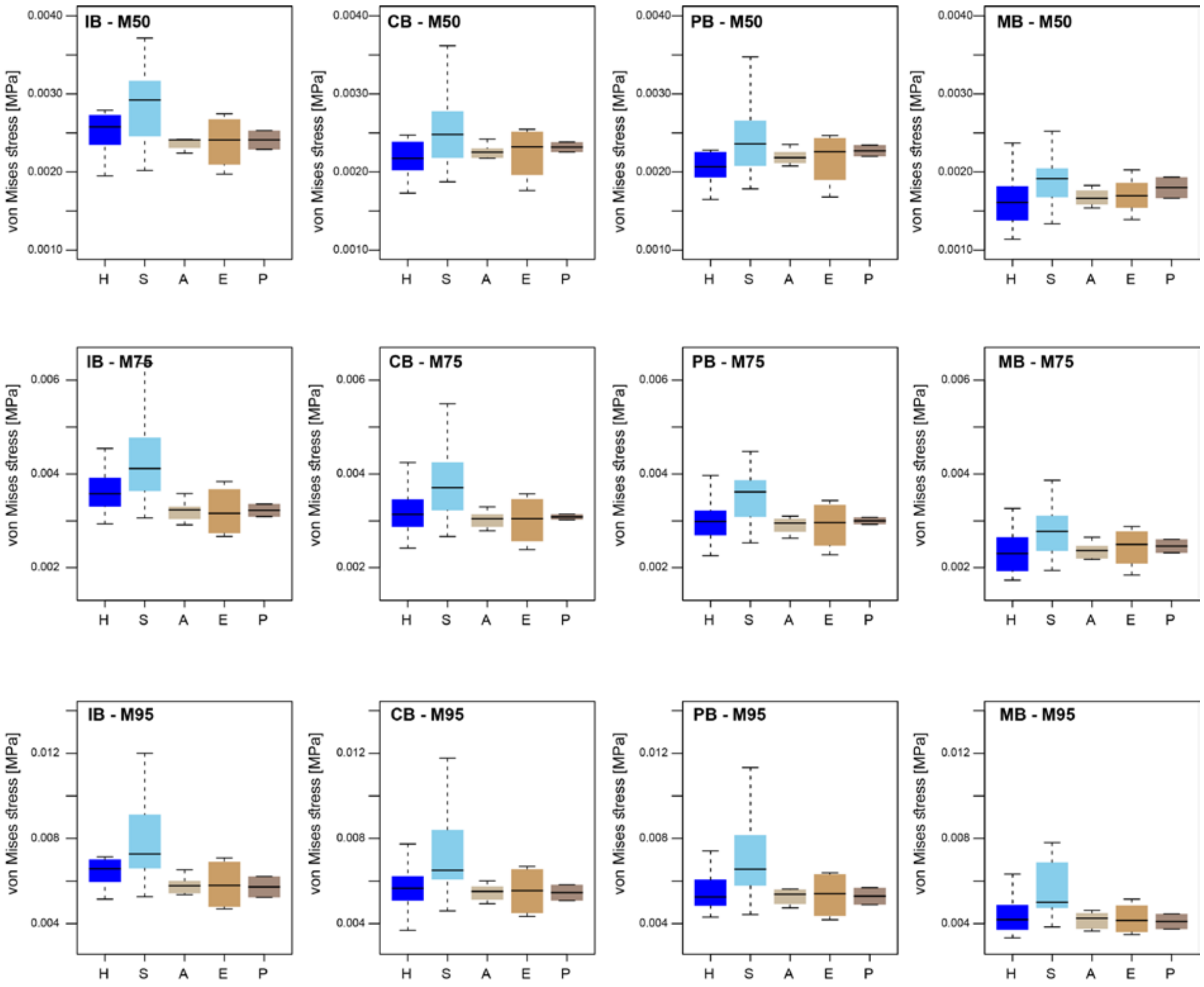

References

1. Kimbel, W. H., White, T. D. & Johanson, D. C. Cranial morphology ofAustralopithecus afarensis: A comparative study based on a composite reconstruction of the adult skull. *Am. J. Phys. Anthropol.* **64**, 337–388 (1984).
2. Skinner, M. M., Gordon, A. D. & Collard, N. J. Mandibular size and shape variation in the hominins at Dmanisi, Republic of Georgia. *J. Hum. Evol.* **51**, 36–49 (2006).
3. Kimbel, W. H. & Rak, Y. The cranial base of Australopithecus afarensis: new insights from the female skull. *Philos. Trans. R. Soc. B Biol. Sci.* **365**, 3365–3376 (2010).
4. Villmoare, B. *et al.* Supplementary Materials for Early Homo at 2.8 Ma from Ledi-Geraru, Afar, Ethiopia. *Science (80).* **347**, (2015).
5. De Ruiter, D. J. *et al.* Mandibular remains support taxonomic validity of Australopithecus sediba. *Science (80).* **340**, 1232997 (2013).
6. Benazzi, S., Kullmer, O., Schulz, D., Gruppioni, G. & Weber, G. W. Individual tooth macrowear pattern guides the reconstruction of Sts 52 (Australopithecus africanus) dental arches. *Am. J. Phys. Anthropol.* **150**, 324–329 (2013).
7. Keyser, A. W. The Drimolen skull: The most complete australopithecine cranium and mandible to date. *S. Afr. J. Sci.* **96**, 189–193 (2000).
8. Leakey, M. G. *et al.* New fossils from Koobi Fora in northern Kenya confirm taxonomic diversity in early Homo. *Nature* **488**, 201–204 (2012).
9. Vekua, A. A New Skull of Early Homo from Dmanisi, Georgia. *Science (80-.).* **297**, 85–89 (2002).
10. Schwartz, J. H., Tattersall, I. & Chi, Z. Comment on ‘A Complete Skull from Dmanisi, Georgia, and the Evolutionary Biology of Early Homo’. *Science (80).* **344**, 360–360 (2014).
11. Lordkipanidze, D. *et al.* A Complete Skull from Dmanisi, Georgia, and the Evolutionary Biology of Early Homo. *Science (80).* **342**, 326–331 (2013).
12. Van Arsdale, A. P. & Lordkipanidze, D. A Quantitative Assessment of Mandibular Variation in the Dmanisi Hominins. *PaleoAnthropology* **2012**, 134–144 (2012).
13. Margvelashvili, A., Zollikofer, C. P. E., Lordkipanidze, D., Peltomaki, T. & Ponce de Leon, M. S. Tooth wear and dentoalveolar remodeling are key factors of morphological variation in the Dmanisi mandibles. *Proc. Natl. Acad. Sci.* **110**, 17278–17283 (2013).
14. Tattersall, I. & Sawyer, G. J. The skull of Sinanthropus from Zhoukoudian, China: a new reconstruction. *J. Hum. Evol.* **31**, 311 (1996).
15. Chih-Wei Hsu, Chih-Chung Chang, and C.-J. L. A Practical Guide to Support Vector Classification. *BJU Int.* **101**, 1396–400 (2008).
16. Decoste, D. & Schölkopf, B. Training Invariant Support Vector Machines. *Mach. Learn.* **46**, 161–190 (2002).
17. Vapnik, V. N. *The Nature of Statistical Learning Theory*. (Springer New York, 2000).
18. Zhang, L., Zhou, W. & Jiao, L. Hidden Space Support Vector Machines. *IEEE Trans. Neural Networks* **15**, 1424–1434 (2004).
19. Ben-Hur, A., Ong, C. S., Sonnenburg, S., Schölkopf, B. & Rätsch, G. Support vector machines and kernels for computational biology. *PLoS Comput. Biol.* **4**, (2008).
20. Schölkopf, B. & Smola, A. J. *Learning with kernel: Support Vector Machines, Regularization, Optimization and Beyond*. *The MIT Press* (2001).
21. Williams, C. K. I. Learning With Kernels: Support Vector Machines, Regularization,

Optimization, and Beyond. *J. Am. Stat. Assoc.* **98**, 489–489 (2003).

22. Kuhn, M. Classification and regression training. *Astrophys. Source Code Libr.* 1–198 (2015).

23. Kuhn, M. & Johnson, K. Measuring Performance in Classification Models. in *Applied Predictive Modeling* 247–273 (Springer, New York, NY, 2013).

24. Marcé-Nogué, J., De Esteban-Trivigno, S., Escrig, C. & Gil, L. Accounting for differences in element size and homogeneity when comparing Finite Element models: Armadillos as a case study. *Palaeontol. Electron.* **19**, 1–22 (2016).
